# Prognosis with Incidental Rolandic Spikes

**DOI:** 10.15844/pedneurbriefs-29-3-2

**Published:** 2015-03

**Authors:** Tracy S. Gertler, Cynthia V. Stack

**Affiliations:** 1 Division of Neurology, Ann & Robert H. Lurie Children's Hospital of Chicago, Chicago, IL; 2 Departments of Pediatrics and Neurology, Northwestern University Feinberg School of Medicine, Chicago, IL

**Keywords:** Cognition, EEG, Epilepsy, Levetiracetam, Rolandic

## Abstract

Investigators from Johns Hopkins University reported a cohort of 27 patients with incidentally-noted rolandic spikes (RS) on EEG.

Investigators from Johns Hopkins University reported a cohort of 27 patients with incidentally-noted rolandic spikes (RS) on EEG. The cohort included children aged 3-9, mostly male, with 19% comorbid ADHD and 19% familial epilepsy. Of 27 patients, 7 developed seizures, including 3 with benign rolandic epilepsy (BRE) and 1 with febrile seizures and learning difficulties. The patient with febrile seizures and 6 additional patients were offered levetiracetam. Of 7 patients given levetiracetam, five reportedly ‘improved’; the remaining 2 were not affected and lost to follow-up. [1]

COMMENTARY. While BRE is defined by characteristic seizures and an EEG with RS, the significance of RS found incidentally is more enigmatic. To begin, the true incidence of this finding may depend on both phenotype and type of EEG. Though the incidence of asymptomatic RS is reported as 2–3% of children aged 5–12 [2], the incidence varies based on psychiatric comorbidities such as autism and ADHD, and the type (i.e. with sleep-deprivation), and duration of EEG performed.

As a diagnosis of BRE confers an additional risk of language and memory deficits, it is tempting to speculate that a child with a similarly abnormal EEG and comparable learning impairments without seizures would benefit from anticonvulsant therapy. As the authors previously described improved speech following treatment of BRE with levetiracetam [3], the same medication is a rational starting point. Yet, we still lack consensus about whether BRE should be treated at all, if seizures remain infrequent, irrespective of concurrent cognitive impairment [4].

This study hints at a larger question, which is whether RS are truly ‘incidental,’ or rather a biomarker for mild cerebral dysfunction. Indeed, it is debated whether characteristics of RS rather than seizure burden itself have a clearer prognostic role in terms of cognitive impairment [5]. A recent sibling-controlled study used neuropsychological testing to investigate cognition in patients with abnormal EEGs compared to siblings without RS; stereotypic language and memory impairments common to both groups suggested an inherited susceptibility independent of the EEG [6]. Similarly, in an analogous subset of patients with GRIN2A mutations, a spectrum of seizures and cognitive difficulties appear to be epiphenomena of the same molecular bias [7].

McNally and Kossoff [1] suggest that RS may underlie cognitive abnormalities, and are thus sufficient when dually present to warrant an empiric trial of levetiracetam. Pending future prospective controlled trials and a better understanding of the gene underlying an inherited susceptibility, an EEG with RS, BRE, and cognitive impairment, the decision to treat remains a clinical judgement.
